# Patient-Reported Outcome Following Operative and Conservative Treatment of Calcaneal Fractures: A Retrospective Analysis of 79 Patients at Short- to Midterm Follow-Up

**DOI:** 10.3389/fsurg.2021.620964

**Published:** 2021-05-28

**Authors:** Patrick Pflüger, Michael Zyskowski, Frederik Greve, Chlodwig Kirchhoff, Peter Biberthaler, Moritz Crönlein

**Affiliations:** Department of Trauma Surgery, Klinikum Rechts der Isar, Technical University of Munich, Munich, Germany

**Keywords:** calcaneus, calcaneal injury, calcaneal fractures, hindfoot fractures, patient reported outcome

## Abstract

**Background:** Fractures of the calcaneus are severe injuries of the hindfoot, mostly resulting from high-energy axial loads, which still present enormous challenges to modern trauma surgery. Possible variables influencing the outcome are the type of fracture, age, and quality of fracture reduction. These might also be factors affecting the self-reported patient outcome, but large studies are still lacking. Therefore, the aim of this study was to analyze the patient-reported outcome of calcaneal fractures following operative and conservative treatment.

**Methods:** All patients suffering from calcaneal fractures between 2002 and 2015 were enrolled in this retrospective analysis. The calcaneal fractures were classified according to Sanders and the AO classification system. For further analysis, two groups were formed: group I involved complex intra-articular fractures defined by the involvement of the posterior calcaneal facet, while group II consisted of extra-articular and process calcaneal fractures. Data were collected *via* the patient registry, radiographs, and a standardized questionnaire (Foot and Ankle Outcome Score, FAOS). For outcome analysis, non-parametric Mann–Whitney *U*-test was performed, and Spearman's rank correlation coefficient was calculated.

**Results:** In total, the functional outcome of 79 patients with calcaneal fractures was analyzed. In group 1 (*n* = 43), the mean FAOS score was 65.5 ± 18.9. The surgically treated patients with a Sanders type II calcaneal fracture had a mean FAOS score of 72.9 ± 17.2, type III fractures had 65.6 ± 20.8, and type IV had 61.1 ± 19 (*p* = 0.15). The reoperation rate was 22%, most frequently caused by wound complications (10%). The mean follow-up time was 64.5 ± 44 months. The mean FAOS score of group 2 (*n* = 36) was 75.2 ± 18.4, and 83% of the patients (=30) were managed conservatively. Only one out of six operatively managed patients had a reoperation due to regular implant removal. The mean follow-up time was 31 ± 25.9 months.

**Conclusion:** Intra-articular calcaneal fractures are severe injuries of the hindfoot leading to a fair to poor functional outcome in the majority of the patients. Complications regarding wound healing are the most common causes for revisional surgery. Extra-articular calcaneal fractures are a heterogenous entity commonly managed non-operatively. Overall, they show a better functional outcome in comparison to intra-articular calcaneal fractures.

## Introduction

Fractures of the calcaneus are severe injuries presenting an enormous challenge to modern trauma surgery care. Although the calcaneus is involved in about 60% of all fractures of the lower extremity, it remains to be of rare incidence accounting for about 2% of all skeletal fractures (1). Up to 75% of all calcaneal fractures are severe intra-articular fractures, involving the subtalar joint (2). Most calcaneal fractures occur in young male patients around the age of 40 (3). Falls from height and motor vehicle accidents are described as typical injury mechanisms in the current literature (4, 5).

The computed tomography (CT)-based Sanders classification system helps to precisely analyze and describe calcaneal fractures, resulting in an adapted treatment plan depending on the severity of the fracture (2, 6).

Nowadays, due to the development of new surgical techniques and implants, displaced intra-articular fractures are more frequently treated surgically (3). However, in the current literature, there is still a lack of broad studies proving that operative treatment is superior to non-operative treatment in these cases (7). Important factors to consider in decision-making regarding the best treatment are age, patient's comorbidities, functional demand, and compliance as well as the extent of soft tissue injury and fracture characteristics (8). For a satisfying outcome, it is essential to achieve adequate fracture reduction along with restoring the anatomical joint congruency (8).

Two reviews including operatively and non-operatively managed calcaneal fractures state a fair long-term outcome (3, 7). The adverse outcome is attributed to postoperative complications and the high probability for late subtalar arthritis (3, 9, 10).

Calcaneal fractures not involving the subtalar joint are often managed conservatively, and only vertical fractures of the tuberosity, displaced medial process, and large anterior process fractures are treated surgically (11). In contrast to complex intra-articular calcaneal fractures, the overall outcome of operative or non-operative managed process fractures of the calcaneus is satisfactory (11–13).

Calcaneal fractures are rare skeletal injuries, often leading to permanent pain and limitations, especially with increasing fractures severity. There are important patient- and injury-specific factors that determine the radiological and functional outcome. These might also influence the self-reported patient outcome, but large studies are still missing. Therefore, the aim of this study was to analyze the patient-reported outcome of calcaneal fractures following operative and conservative treatment.

## Methods

### General Data

After ethical board approval (no: 409/15 S, Technical University of Munich), the retrospective cohort study was conducted between 2002 and 2015 in a level I trauma center. All patients presenting with calcaneal fracture at the department of trauma and orthopedic surgery were reviewed for enrollment. Inclusion criteria: patients of 18 years and older who were capable of giving informed consent and had suffered a calcaneal fracture Sanders type I–IV or AO-83A-C as well as fractures of the anterior process of the calcaneus or of the sustentaculum tali. The outcome of conservatively and operatively managed patients was analyzed. Patients with a fracture due to malignancy, an active malignancy, substance abuse, presenting for revision surgery after external operation, and with legal guardian were excluded.

### Surgical Technique and Rehabilitation

A board-certified trauma surgeon specialized in lower extremity surgery evaluated indications for surgery. Patients with a displaced intraarticular calcaneal fracture (Sanders type II-IV) were indicated for operative treatment. Only in cases where operative treatment was unfeasible due to multiple comorbidities or limited function (e.g., wheelchair, bedridden), intraarticular fractures were managed conservatively. Operative treatment involved open reduction and internal fixation (ORIF) with screw-, plate- or K-wire osteosynthesis. Surgery was performed after soft tissue consolidation. In intra-articular fractures the patient was placed in a lateral position, prophylactic cefuroxime 1.5 g was administered and a thigh tourniquet applied and inflated, if necessary. Usually, preliminary fixation was achieved via an extended lateral approach with the help of K-wires and definitely secured with a locking compression plate. Only in individual cases a minimal invasive approach for screw osteosyntheses was performed.

Following open reduction and internal fixation (ORIF), the patients had to partially weight bear for 6 weeks with a walking boot. Patients with conservative treatment had to partially weight bear for 6 to 8 weeks with a walking boot and undergo regular radiographic evaluations. After 6 to 8 weeks and proper radiographic follow-ups, full weight bearing without the walking boot was allowed.

### Radiological Analysis

Calcaneal fractures were classified based on CT images using the Sanders as well as the AO classification system. According to the fracture classification, two groups were formed: group 1 involved complex intra-articular fractures defined by the involvement of the posterior calcaneal facet, and group 2 consisted of extra-articular and process calcaneal fractures including type A fractures according to the AO classification, fractures of the anterior process of the calcaneus, or of the sustentaculum tali. The quality of surgery was evaluated by the postoperatively restored Bohler's angle. Studies showed that restoration of Bohler's angle can be crucial to obtain satisfactory results in displaced intra-articular calcaneal fractures (14, 15).

**Figure 1:**
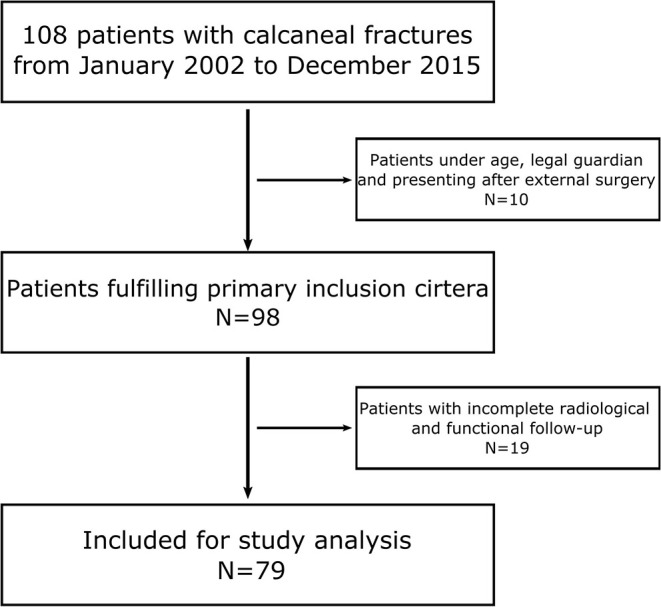
Flow diagram demonstrating the patients included for the study analysis.

Only patients with a minimum radiological follow-up time of 3 months were included for further analysis. Radiographs and, if available, CT were systematically analyzed to determine the fracture type using the Sanders as well as the AO classification. In all patients, the Bohler's (standard: 25–40°) and the Gissane (standard: 120–140°) angles were measured at the time of fracture (1). In the surgically treated patients, these angles were also determined after surgery (see Figure 1). The absolute difference between pre- and postoperative angles was calculated.

### Functional Outcome

For the evaluation of the functional outcome following calcaneal fractures, the Foot and Ankle Outcome Score (FAOS) was used. The FAOS is a self-administered patient-relevant outcome questionnaire consisting of 42 items (range: 0–100). The German version of the FAOS is a valid and reliable instrument for foot and ankle patients (16). The FAOS results were graded as “excellent” (100–90), “good” (89–80), “fair” (79–70), and “poor” (<70) as adapted from the evaluation of Thordarson and Krieger (17). After hospital treatment and standard postoperative visits, patients were invited by letter to complete the FAOS. The minimum follow-up for the patient-reported outcome was 12 months.

While general data such as age, gender, affected side, accident type, hospital admission rate, and date of latest follow-up were collected in both groups, additional information on American Society for Anaesthesiologists (ASA) score, time between fracture and surgery, implants, and reoperation rate were only collected in the surgically treated group.

### Statistics

Data was presented as mean ± standard deviation (SD). RStudio [RStudio Team (2020). RStudio: Integrated Development Environment for R. RStudio, PBC, Boston, MA URL http://www.rstudio.com/] was used for data processing.

The non-parametric Mann–Whitney *U*-test was used to assess significant differences between two groups, assuming no normally distributed data. To assess the correlation between two variables, Spearman's rank correlation coefficient was calculated. We performed a multiple linear regression model when two or more independent variables influenced the outcome. The results of the inferential analysis are presented as 95% confidence intervals. *P*-value < 0.05 was considered statistically significant.

## Results

**Figure 2:**
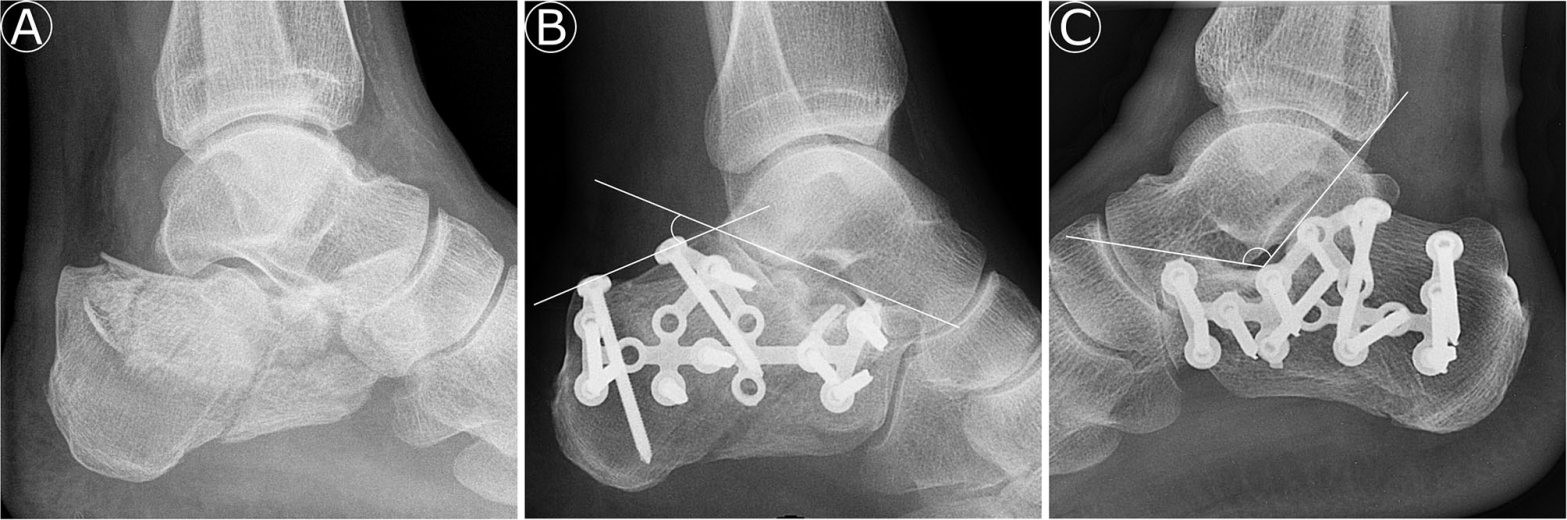
**(A)** Lateral preoperative radiograph of a Sanders-type 3AC calcaneal fracture. **(B)** Lateral postoperative radiograph of the calcaneal fracture from **(A)** with improved plotted Bohler's angle (39°). **(C)** Lateral postoperative radiograph of a calcaneal fracture with a marked Gissane angle of 123°.

Between January 1, 2002 and December 31, 2015, 108 patients were treated at the department of trauma and orthopedic surgery for a calcaneal fracture. In total, 79 patients (73%) were available for further data analysis (Figure 2). The mean age was 54 ± 17 years, including 24 female and 55 male patients. In 40 patients (51%), the right side was injured, whereas 39 (49%) patients fractured their left calcaneus.

### Complex Intra-Articular Fractures

#### General Data

In group 1, 43 patients were enrolled, with a mean age of 48 ± 17 years, including eight female (19%) and 35 male (81%) patients (see Table 1). In 22 patients (51%), the right side was injured, whereas 21 (49%) patients fractured their left calcaneus. The injury mechanism was in 34 cases a fall from height (>1 m), in five cases traffic accidents, in two cases contusions of the foot, and in two cases no further information on injury mechanism was available. The mean ASA score of the operated patients was 1.6 ± 0.6. The mean follow-up time was 67 ± 45 months.

**Table 1:** Overview of general data and functional outcome of patients with complex intra-articular and extra-articular/process fractures of the calcaneus.

		**Complex intra-articular fractures**	**Extra-articular and process fractures**
**Age (year)**			
	Mean and standard deviation	48 ± 17	44 ± 17
	Minimum	18	19
	Maximum	79	76
**Sex**			
	Female	19% (*n* = 8)	44% (*n* = 16)
	Male	81% (*n* = 35)	56% (*n* = 20)
**Type of fracture**			
**Sanders**			
II	Type A (AO)	42% (*n* = 18)	20% (*n* = 7)
III	Proc, ant. Calc.	37% (*n* = 16)	72% (*n* = 26)
IV	Sustentaculum	21% (*n* = 9)	–
	Other		8% (*n* = 3)
**Treatment**			
	Operation	86% (*n* = 37)	17% (*n* = 6)
	Conservative	14% (*n* = 6)	83% (*n* = 30)
**Foot and ankle outcome score**			
	Mean and standard deviation	67 ± 18.3	75 ± 18.4
	Minimum	23	32
	Maximum	96	94

#### Radiological Analysis

Regarding the Sanders classification, 18 patients had a Sanders type II, 16 a Sanders type III, and nine a type IV fracture. A total of 37 patients (86%) were treated operatively after a mean of 6 ± 4.3 days. In the conservatively treated group, four patients had a Sanders type II and two a Sanders type III fracture.

The mean preoperative Bohler's angle accounted for 19 ± 8.9° (95% CI, 17.1–22.4), and the Gissane angle was 112 ± 12.8° (95% CI, 110.3–114.2). Postoperatively, the mean Bohler's angle was 29 ± 8.5° (95% CI, 27.2–32.2), and the Gissane angle accounted for 117 ± 10.8° (95% CI, 115.5–119). Due to surgery, the Bohler's angle improved by 11 ± 7.9° (95% CI, 9.1–14.6) and the Gissane angle by 13 ± 10.5° (95% CI, 10.4–16.5). The extent of improvement of Bohler's angle corrected for age and type of fracture (Sanders classification) did not significantly influence the FAOS score (*p* = 0.57). Patients with a preoperative Bohler's angle <15° demonstrated no adverse outcome (*p* = 0.72).

#### Functional Outcome

**Figure 3:**
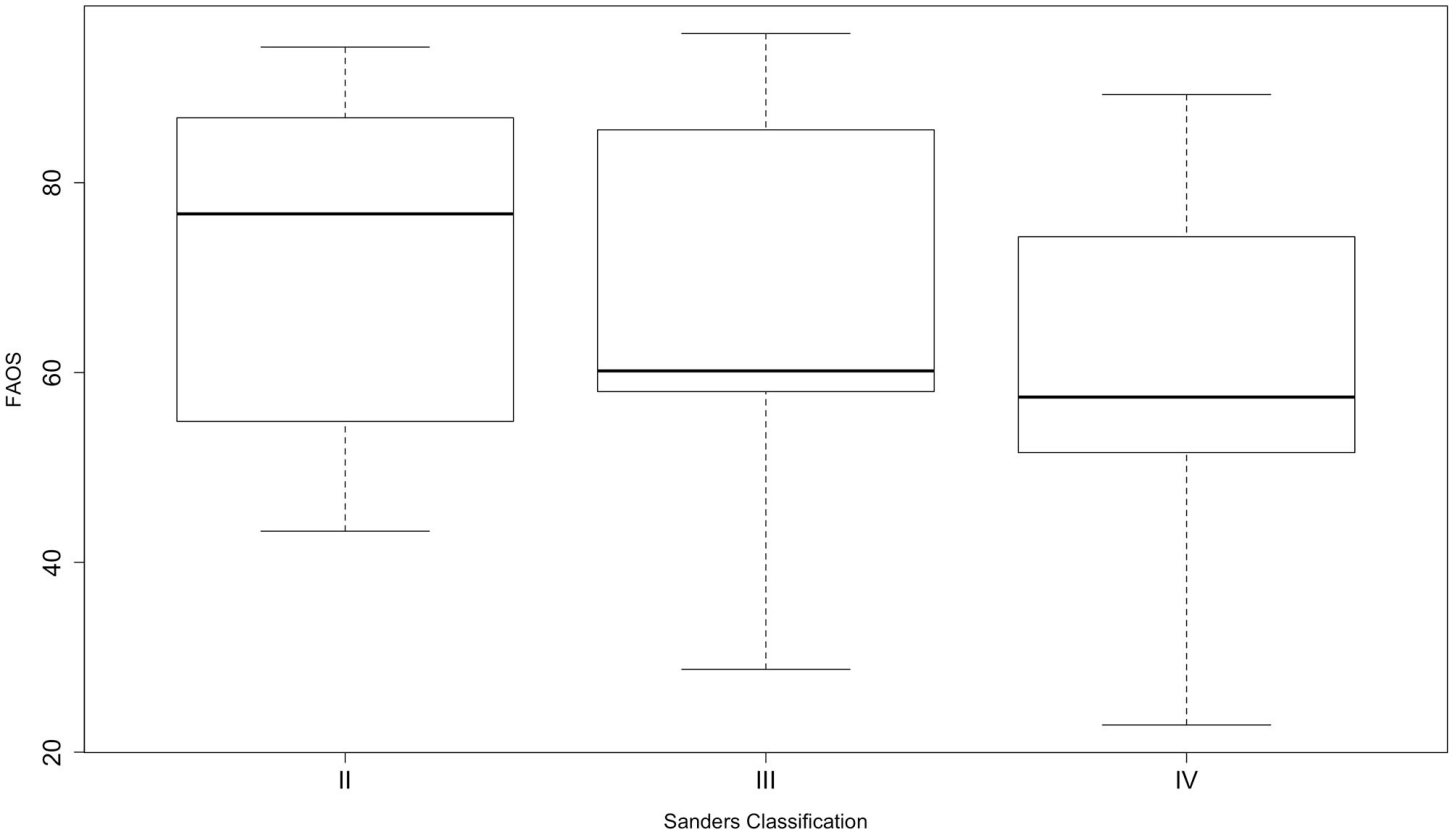
Boxplots of foot and ankle outcome score among the different Sanders-type fractures of surgically treated patients. The whiskers show the minimum and maximum.

The overall mean FAOS score was 67 ± 18.3 (95% CI, 61–72), with five “excellent” (12%), eight “good” (19%), four “fair” (9%), and 26 “poor” (60%) results. The surgically treated patients with a Sanders type II calcaneal fracture had a mean FAOS score of 73 ± 17.2 (95% CI, 64–80.9), type III fractures presented a FAOS score of 66 ± 20.8 (95% CI, 53.9–77.4), and type IV fractures showed a score of 61 ± 19 (95% CI, 49.9–72.4) (Figure 3). Regarding the FAOS score, no significant differences were found between surgically treated Sanders-type fractures (*p* = 0.23). The age of the patient adjusted to the type of fracture (Sanders classification) did not significantly influence the FAOS score (*p* = 0.39). There was also no difference between operatively (*n* = 14) and conservatively (*n* = 4) treated Sanders type II fractures (*p* = 0.36). Patients requiring revision surgery had a mean FAOS of 62 ± 19.45 (95% CI, 49.4–74.8).

#### Reoperation, Implants, and Hardware Removal

Eight of 37 patients (22%) needed revision surgery, whereas five cases had complications with wound healing (14%) and one patient suffered from implant failure (3%). One patient developed a tarsal tunnel syndrome (3%) and another one had arthritis of the subtalar joint with following subtalar arthrodesis 4 years after the initial trauma. The wound healing complications occurred in four Sanders type III and another one in a type II fracture. The subtalar arthrodesis was the consequence of Sanders type IV fracture. Patients requiring revision surgery did not have a significantly lower FAOS score (*p* = 0.52).

In 73% (*n* = 27) of the cases, locking compression plate osteosynthesis was performed, and 8% (*n* = 3) received non-locking plate osteosynthesis, whereas in 16% (*n* = 6) of the cases screws were used (Table 2). The extended lateral approach was used for the locking compression and non-locking plates (*n* = 30); screws were inserted through a stab incision under radiological guidance (*n* = 3) and additionally under arthroscopic visualization of the subtalar joint (*n* = 3). The percutaneous locking plate was implanted *via* a sinus tarsi approach. After bone consolidation, the implant was removed in 54% (*n* = 20) of the patients after a mean of 14 ± 3.4 months.

**Table 2:** Surgically treated complex intra-articular calcaneal fractures with the distribution of implants among the different Sanders-type fractures.

	**Sanders II**	**Sanders III**	**Sanders IV**
LCP	8	11	8
Non-locking plate	–	2	1
Percutaneous plate	1	–	–
Screws	5	1	–

*LCP, locking compression plate*.

### Extra-Articular and Process Fractures

#### General Data

In total, 36 patients matched our inclusion criteria for this cohort. The mean age was 44 ± 17 years, including 16 female (44%) and 20 male (56%) patients (Table 1). In 18 patients (50%), the right side was injured, whereas the other 18 (50%) fractured their left calcaneus. Six patients (17%) were treated operatively after a mean of 6 ± 4.9 days. Three patients had a type A2 fracture, and another three patients showed a fracture of the anterior process of the calcaneus. The remaining 30 patients were treated conservatively. The mean ASA score of the operated patients was 1.5 ± 0.5. The follow-up time was 31 ± 25.9 months.

#### Radiological Analysis

Regarding the classification, seven patients had a type A fracture according to the AO classification, while 26 had a fracture of the anterior process of the calcaneus and three other fractures (two stress fractures, only visible in MRI scans and one fracture of the sustentaculum tali).

The mean Bohler's angle was 33 ± 5.9° (95% CI, 30.8–34.7), and the Gissane angle accounted for 118 ± 10.3° (95% CI, 114.7–121.5).

#### Functional Outcome

The mean FAOS score was 75 ± 18.4 (95% CI, 69.2–81.2), with nine “excellent” (25%), nine “good” (25%), seven “fair” (19%), and 11 “poor” (31%) results. The type A fractures had a mean FAOS score of 86 ± 7 (95% CI, 80.6–90.9). There was no difference between operatively and conservatively treated type A fractures (*p* = 0.57). The mean FAOS score of patients with fractures of the anterior process of the calcaneus was 73 ± 19.3 (95% CI, 65.8–80.6). The surgically treated patients (*n* = 3) had a FAOS score of 50 ± 15.5 (95% CI, 26.2–74.5), and the non-operatively treated patients (*n* = 23) had a score of 76 ± 17.6 (95% CI, 68.9–83.5). We found no correlation between age and FAOS score in the group of type A fractures and fractures of the anterior process of the calcaneus (*p*_A_ = 0.45; *p*_P_ = 0.50).

#### Reoperation, Implants, and Hardware Removal

Patients with an anterior process fracture and operative treatment, received two times a K-wire osteosynthesis and once screw osteosynthesis was performed. Only one patient had a planned K-wire removal 3 months postoperatively. In the case of type A fractures, screw osteosynthesis was performed two times and once in combination with plate osteosynthesis.

## Discussion

Overall, the functional outcome of 79 patients including 43 complex intra-articular and 36 extra-articular or process fractures of the calcaneus treated between 2002 and 2015 was assessed. The majority of patients with intra-articular calcaneal fractures only reached “poor” results, and the functional outcome was not significantly influenced by age or Sanders classification. Sanders type IV fractures tended to have the lowest FAOS score, but the difference between Sanders type fracture did not reach a level of significance. Wound infections were the most common cause for reoperation in this subgroup. Patients with an extra-articular or process fracture of the calcaneus were mostly treated non-operatively and stated a better self-reported outcome.

Patients with an extra-articular or process fracture of the calcaneus reached a mean FAOS of 75. The studies in the literature involving extra-articular or process fracture of the calcaneus were solely retrospectively performed with a small number of patients. Consequently, there is still missing evidence concerning the optimal therapy regimen in these cases. The fractures are mainly treated conservatively, and only in displaced fractures is operation indicated (8, 12, 18, 19). Overall, studies reported a satisfactory outcome after conservatively or operatively treated extra-articular calcaneal fractures (11, 12, 20), but inadequately treated tuberosity avulsion fractures can lead to limitations involving the Achilles tendon and, in case of anterior process fractures, to painful non-unions (11).

The mean age of included patients suffering from intra-articular calcaneal fractures was 48 years, with a preponderance of male patients. In the majority of the cases, a fall from height was the injury mechanism. In comparison to other studies in the literature, the demographic data shows a typical age and gender distribution for calcaneal fractures since these fractures are often seen in young males with high-energy trauma (5, 21). The majority of these patients were treated operatively after a mean time of 6.5 days. This is in line with the trend toward a surgical approach over the last two decades (8). Operative treatment can be advantageous depending on patient factors, the soft tissue conditions, and fracture characteristics (3). Since soft tissue management is essential in calcaneal fractures, the swelling should be minimized before surgery is performed (2). This usually occurs within 10 days according to current literature (6). A delay to surgery over 14 days is associated with a greater risk of complications (22–24).

Other studies analyzing the treatment of displaced intra-articular calcaneal fractures report functional outcome scores ranging from 68 to 84 points regarding the American Orthopaedic Foot and Ankle Society (AOFAS) hindfoot score (9, 14, 24, 25). The AOFAS score consists of physical examination, reported function, self-reported pain items. Its validity and reliability have been criticized in the literature before (26, 27). The slightly inferior score, on average, might be due to the study design using a solely patient-reported outcome measurement tool. In contrast to the AOFAS, the FAOS includes items for activities of daily living, sport and recreational activities, and foot- and ankle-related quality-of-life items. Due to the severity of the injury, low self-reported ratings in these categories are plausible, leading to an overall lower score. In general, there is a slight trend toward patient-reported outcome measurement tools since they provide benefits regarding safety, effectiveness, and experience (28). Patients become involved by directly assessing their symptoms, disability, and quality of life, thereby avoiding observer bias. To date, the presented work reports about the largest patient population suffering from calcaneal fractures using a self-reported patient outcome measurement questionnaire to assess and analyze functional outcome.

The analysis show no relation between age, Sanders classification, and functional outcome score in the patient population. These findings are in accordance with a recently published study of Evers et al., who found no association between age and functional outcome in intra-articular surgically treated calcaneal fractures (24). In contrast to these findings, Buckley et al. showed, in a subgroup analysis of a randomized control trial, that patients younger than 29 years scored significantly higher on the scoring scales after surgery of a displaced intra-articular calcaneal fracture compared to conservative treatment (29), but two other studies reporting on 44 and 33 patients found satisfactory outcome results of patients older than 65 years (30, 31). The mentioned controversial studies show that the functional outcome cannot be traced down to only the age of the patients. In fact, multiple other factors, such as co-morbidities, have to be considered. Peripheral vascular disease, poorly controlled diabetes, and smoking habits are independent risk factors for an adverse outcome (10, 32).

There was no significant difference between Sanders fracture types treated surgically regarding the functional outcome. Sanders et al. stated in a study in 2014 that the Sanders classification system still remains prognostic and that severe fractures were more likely to develop an adverse outcome (6), which is in line with the results of the FAOS among the different Sanders type fractures with a trend to a worse outcome with increasing severity without reaching a level of significance. Other studies also reported that Sanders type II fractures lead to better results than type III and IV calcaneal fractures (9, 33), but there are also studies that criticize the classification system due to a low intra- and interobserver reliability (34, 35).

The presented data did not show a significant difference between operatively and non-operatively treated Sanders type II fractures. This is in some way not surprising considering that long-term results of surgically treated calcaneus fractures only show fair results and a review published by Luo et al. came to the conclusion that there is no certainty about the superiority of the operative treatment for displaced intra-articular calcaneal fractures (7, 36). But there are two important factors to consider in this context: first, the retrospective study design and the small number of Sanders type II fractures which were managed non-operatively. Second, the long-term studies and reviews included analysis from the 1990s; since then, new and allegedly better techniques (e.g., minimally invasive) for open reduction and internal fixation were developed.

In the patient cohort, both the Bohler's and the Gissane's angle improved postoperatively; however, the relation between the improvement of Bohler's angle, Sanders classification, and the functional outcome was not significantly different. Several studies support this observation, showing no correlation between Bohler's angle and the final functional outcome (37–39). The only correlation between Bohler's angle and outcome might be too simple since different factors influence the outcome. The congruity of the articular surface of the posterior facet, only visualized on CT scans, especially seems to significantly influence the final outcome (40, 41).

The 22% re-operation rate, which was mostly caused by wound infections, is in line with the current literature considering that mainly an extended lateral approach was used in the presented study (3, 37). New minimally invasive techniques, e.g., the sinus tarsi approach, can reduce wound infections compared to an extended lateral approach in displaced intra-articular fractures, whilst the functional outcome showed comparable results (42). A review analyzing eight case series of displaced intra-articular calcaneal fractures treated with the sinus tarsi approach came to the conclusion that well-designed randomized trials are needed to clearly prove superiority (43). Studies are awaited to demonstrate definite indications for a limited lateral approach in displaced intra-articular calcaneal fractures, leading to a satisfactory outcome.

The study results clearly show that the treatment of intra-articular calcaneal fractures calls for improvements in patient care since self-reported outcome is often poor. In intra-articular calcaneal fractures, operative treatment was mainly performed *via* an extended lateral approach and revision surgery mostly caused by wound complications. In clinical practice, operative treatment needs to focus on a surgical approach with minimal soft tissue damage and optimal reduction with the help of adjacent modern tools to fully restore the congruity of the articular surface of the posterior facet.

The results of the presented study may be influenced by several potential limitations. Due to its retrospective design, there was no randomization of the patients, and no *a priori* sample size calculation was performed. The solely patient-reported outcome might distort the final results since the FAOS score includes more categories representing activity and participation. Furthermore, long-term clinical and radiological results were not part of the study, and radiographs were taken for clinical follow-up and not for research purposes in a strictly standardized manner. The reported posttraumatic arthritis rate needs to be interpreted with caution. The surgical procedures evaluated in this study were performed between 2002 and 2015, and especially in regard to the first years, the type of implants and surgical management evolved over the years. However, to the authors' knowledge, the study differs from the previously published studies in terms of providing current evidence regarding the patient-reported outcome following the operative and conservative treatment of fractures to the calcaneus.

## Conclusions

Intra-articular calcaneal fractures are severe injuries of the hindfoot, leading to a fair to poor functional outcome in the majority of the patients. Complications regarding wound healing are the most common causes for revisional surgery. Extra-articular calcaneal fractures are a heterogenous entity commonly managed non-operatively. Overall, they show a better functional outcome in comparison to intra-articular calcaneal fractures.

## Data Availability Statement

The raw data supporting the conclusions of this article will be made available by the authors, without undue reservation.

## Ethics Statement

The studies involving human participants were reviewed and approved by Ethical board Technical University of Munich. The patients/participants provided their written informed consent to participate in this study.

## Author Contributions

PP and MC analyzed the data and wrote the paper. MZ and FG performed investigation and data curation. PB, CK, and MC designed the study. All authors contributed to the article and approved the submitted version.

## Conflict of Interest

The authors declare that the research was conducted in the absence of any commercial or financial relationships that could be construed as a potential conflict of interest. The reviewer LM declared a shared affiliation, with no collaboration, with the authors PP, MZ, FG, CK, PB, and MC to the handling Editor.
